# Same Dosages of rPRV/XJ5-gI^−^/gE^−^/TK^−^ Prototype Vaccine or Bartha-K61 Vaccine Similarly Protects Growing Pigs Against Lethal Challenge of Emerging vPRV/XJ-5 Strain

**DOI:** 10.3389/fvets.2022.896689

**Published:** 2022-07-01

**Authors:** Qinghai Ren, Lin Li, Haochun Pan, Xiaobo Wang, Qingqing Gao, Changchao Huan, Jin Wang, Wei Zhang, Luyao Jiang, Song Gao, Yan Kai, Changhai Chen

**Affiliations:** ^1^Key Laboratory of Avian Bioproducts Development, Ministry of Agriculture and Rural Affairs, Yangzhou University, Yangzhou, China; ^2^Jiangsu Co-innovation Center for Prevention and Control of Important Animal Infectious Diseases and Zoonoses, Yangzhou University, Yangzhou, China; ^3^Institutes of Agricultural Science and Technology Development, Yangzhou University, Yangzhou, China; ^4^College of Veterinary Medicine, Yangzhou University, Yangzhou, China; ^5^Jiangsu Provincial Center for Animal Disease Control and Prevention, Nanjing, China

**Keywords:** pseudorabies, variant pseudorabies virus, Bartha-K61 vaccine, rPRV/XJ5-gI^−^/gE^−^/TK^−^ prototype vaccine, efficacy, growing pigs

## Abstract

Variant pseudorabies viruses (vPRV) have constantly emerged in China since late 2011. In the present study, a 1 × 10^6.0^ TCID_50_ per-animal dosage of a commercially available Bartha-K61 vaccine and an rPRV/XJ5-gI^−^/gE^−^/TK^−^ prototype vaccine freshly extracted from the vPRV/XJ-5 at the same dose were administered to evaluate the immune effectiveness thereof on growing pigs to prevent lethal strikes caused by vPRV/XJ-5. The results suggest that the Bartha-K61 vaccine at a dose of 1 × 10^6.0^ TCID_50_ per animal and the same dosage of the rPRV/XJ5-gI^−^/gE^−^/TK^−^ prototype vaccine protected growing pigs against the lethal challenge of vPRV/XJ-5 strain with 100% survive rate. Furthermore, the outcome of the clinical score, virus shedding, weight gain, and viral loads in different pig tissues in these two groups demonstrates that either the Bartha-K61 vaccine or the rPRV/XJ5-gI^−^/gE^−^/TK^−^ prototype vaccine at the same dose exhibited parallel efficacy in pigs against the lethal challenge with the XJ-5 strain of vPRV.

## Introduction

Pseudorabies virus (PRV) or Suid herpesvirus 1 (SHV-1), which are members of the *Alphaherpesvirinae* subfamily of the *Herpesviridae*, trigger Aujeszky's disease, which is also referred to as pseudorabies (PR) (1). There is currently only one known category of serotype (1–3). Even though PRV can affect dogs, cats, cattle, sheep, rabbits, and other mammal species, pigs have been found to be suitable natural hosts and latent carriers for PRV (4). While field isolates possibly have a different virulence from other strains, both induce abortions in pregnant sows and spread deadly infection in piglets. PRV resistance in piglets is age-dependent, and they may be susceptible to deadly infections with the characteristics of neuronal signs, such as sudden death, convulsions, and ataxia; and animals older than 1 year are more likely to manifest respiratory distress and subclinical infection. In pregnant animals, infection of fetuses often leads to resorption, mummification, or abortion (5).

As this is an attenuated vaccine strain that has been deprived of the entire gE gene and the partial gI gene, Bartha-K61 is undoubtedly conducive to PRV control and eradication (6–8). By using the gE Enzyme-Linked Immunosorbent Assay (ELISA) serologic test in combination with Bartha-K61 vaccine, PRV was eradicated in Canada, the US, and certain European countries over the last several decades (9). In China, PRV was acknowledged by the general public for the first time in 1947, then it was subsequently employed extensively in several provinces (10). To prevent PRV outbreak, China imported the Bartha-K61 vaccine strain from Hungary in 1979, and PRV was effectively restrained from the 1990s to 2010 (11). In October 2011, however, a PR pandemic broke out among swine herds that had been vaccinated with Bartha-K61 and was rapidly transmitted to several Chinese provinces, thereby significantly hampering the progress of the pig industry (12). A viral genome analysis revealed that the increasing number of PRV variants represent an independent cluster within the phylogenetic tree that are different from typical PRV strains in terms of their antigenicity; importantly, they may induce mortality (10– 30%) in growing or finishing pigs (12). According to the above results, the Bartha-K61 vaccine may not offer sufficient protection against PRV variants (12).

According to the studies involving vPRV prototype vaccines performed in China, the efficacy between attenuate vPRV prototype vaccine based on triple gI/gE/TK deletions and commercial Bartha-K61 vaccine at the same dose in pigs has not been explored until now. Because pigs have been shown to have a pronounced age resistance against PRV, it should be noted that only 3– 4-week-old piglets were employed for the vaccine efficacy comparison in the aforementioned research (13), and growing pigs older than 12 weeks are rarely used for this purpose.

A vPRV strain was previously isolated in Jiangsu Province, China; identified and named the vPRV/XJ-5 strain in 2014; and attenuated by the deletion of gI, gE, and TK genes to develop a prototype vaccine strain rPRV/XJ5-gI^−^/gE^−^/TK^−^ (14). Besides, 12-week-old PRV/gB and PRV/gE antibody-free growing pigs were immunized with either Bartha-K61 or rPRV/XJ5-gI^−^/ gE^−^/ TK^−^ prototype vaccine at a dose of 1 × 10^6.0^ TCID_50_ per animal. Four weeks after immunization, lethal challenge was implemented with vPRV/XJ-5 strain at a dose of 4 × 10^6.0^ TCID_50_ per animal. This study evaluated the efficacy of these two vaccines against vPRV lethal challenge in 16-week-old growing pigs.

## Materials and Methods

### Cells, Viruses, and Commercial Vaccine

Amphotericin B (0.75 μg/mL, Sangon Biotech, China), streptomycin (50 U/mL), penicillin (50 U/mL), and FBS (fetal bovine serum, 5%, Gemini, USA) were added to a Dulbecco's modified Eagle's medium (DMEM; high glucose, Sigma, USA), which was suitable for maintaining Vero cells (ATCC®CCL-81™) when the temperature is 37°C and the carbon dioxide is 5%. For this study, a vPRV/XJ-5 strain in brain tissues from a dead piglet aged 21 days at a farm in Jiangsu Province, China was isolated. There was an outbreak of infectious disease in 2014 that led to the mortality of newborn piglets accompanied by neurologic symptoms (i.e., convulsion, trembling, paralysis, and ataxia) at a farm (15). Here, rPRV/XJ5-gI^−^/gE^−^/TK^−^ refers to a prototype vaccine derived from the vPRV/XJ-5 strain with gI, gE, and TK gene deletions (14).

A previous study reported that stock viral solutions for vaccination-challenge experimentation were accomplished in Vero cells (15). To briefly summarize, at an infection multiplicity of 0.1, vPRV/XJ-5 or rPRV/XJ5-gI^−^/gE^−^/TK^−^ was employed to infect confluent Vero cells in a dish with a 100-mm diameter. One hour after inoculation, 0.01 M PBS (phosphate-buffered saline) with a pH range of 7.2– 7.4 was used to wash the monolayer three times, then the washed monolayer was added with a 10 mL fresh DMEM. In the event that an overt cytopathogenic effect (CPE) was observed, the dish needed to be subjected to three rounds of freezing and thawing in turn. At the same time, a 10-min centrifugation at 3,000 × g was performed to filter out cell debris, and the supernatant liquid was used as the viral stock. In accordance with Reed and Muench (16), a determination of TCID_50_/mL was accomplished following titrations of Vero cells. The Bartha-K61 vaccine (1 × 10^6.0^ TCID_50_ per animal) was purchased from Laboratorios Hipra, SA in Spain.

### Animals, Housing, and Experimental Design

For the present study, 20 healthy 11-week-old Duroc × Landrace × Yorkshire (DLY) hybrid growing pigs were procured from a backyard pig farm in Shandong Province, China. The purchased pigs were unaffected by infections of PRV, the porcine reproduction and respiratory syndrome virus, porcine circoviruses 2 or 3, or the classical swine fever virus, all of which were identified by a serologic test, reverse transcription PCR (RT-PCR), or PCR, as described by Mettenleiter (9). Using ELISA kits to test for PRV/gB and PRV/gE antibodies (IDEXX Laboratories, USA), it was determined that these pigs were derived from sows that were not immune to PRV and were seronegative for PRV. The ears of all the sample pigs were tagged, then the animals were randomly divided into four equal groups (*n* = 5 per treatment group and mock group). In the first week of the experiment, pigs in each group were placed in an isolate room to help them acclimatize. Each pig was given free access to water and identical feed throughout the entire experimental period. The animal protocols were approved by the Animal Care and Use Committee of Yangzhou University (approval ID: SYXK [Su] 2007– 0005).

### Vaccination and Challenge

Pigs in two groups were injected *via* the neck muscle (i.m.) based on the vaccine dose at 12 weeks of age: rPRV/XJ5-gI^−^/gE^−^/TK^−^ prototype vaccine (1 × 10^6.0^ TCID_50_ per animal) or Bartha-K61vaccine (1 × 10^6.0^ TCID_50_ per animal, Laboratorios Hipra, SA, Spain). The pigs in the third group (*n* = 5) were inoculated with the same volume of DMEM, and these animals were challenged with vPRV/ XJ-5 strain i.m. and represented the “challenge alone” group. The remaining five pigs were only inoculated with DMEM i.m. to serve as the “mock” group. When the pigs were 16 weeks old, the challenge-alone and vaccinated pigs were administered an intranasal challenge using the vPRV/XJ-5 strain (4 × 10^6.0^ TCID_50_ per pig), as shown in Table 1.

**Table 1:** Grouping and treatments in different groups.

**Group**	**Vaccine**	**Vaccine dose (TCID_**50**_/pig)**	**Week-old for vaccination**	**Challenge strain**	**Week-old for challenge intranasally**	**Challenge dose (TCID_**50**_)**
Bartha-K61	Bartha K61	1 × 10^6.0^	12	vPRV/XJ-5	16	4 × 10^6.0^
rPRV/XJ5-gI^−^/gE^−^/TK^−^	rPRV/XJ5-gI^−^/gE^−^/TK^−^	1 × 10^6.0^	12	vPRV/XJ-5	16	4 × 10^6.0^
Challenge- only group	-	-	-	vPRV/XJ-5	16	4 × 10^6.0^
Mock group	-	-	-	-	-	-

### Assessment of Clinical Symptoms and Sample Acquisition

On days 1–7 following challenge, oropharyngeal and rectal swabs were sampled from every pig, and we recorded the animals' rectal temperatures and clinical symptoms once a day for 14 consecutive days post-challenge (d.p.c.) (17). Pig weights were measured at 0, 3, 7, 14 d.p.c. We applied the following standards to assess the clinical outcomes of the challenged pigs: (1) one point: increased body temperature within the range of 40– 41°C; (2) two point: fever that exceeded 41°C accompanied by difficult breathing; (3) three point: convulsions; (4) four point: ataxia; and (5) five point: dead or moribund.

Euthanasia and necropsy were performed on the living pigs at 14 d.p.c. After a veterinary pathologist assessed the gross pathological lesions, histopathological examination was conducted on the chosen tissues.

### Enzyme-Linked Immunosorbent Assay

Blood was sampled during the experiment at 0, 7, 14, 21, 28, 35, and 42 days post-vaccination (d.p.v.). After the serum was coagulated, it was isolated through centrifugation, and ELISA kits (IDEXX Laboratories, USA) were employed to discern PRV-specific gE and gB antibodies according to the manufacturer's directions. To briefly summarize, we added 100 μL/well standard serum (dilution, 1:2) and 100 μL/well serum into the plate for a 1-h incubation period at 37°C. After rinsing with buffer five times, horseradish peroxidase (HRP)-conjugated goat anti-pig IgG was also supplemented, followed by an additional 30-min incubation period at 37°C. The sections were rinsed again and further incubated with 100 μL/well 3, 3′, 5, 5′-Tetramethylbenzidine (TMB) substrate solution in the dark for an additional 15 min. We then added 50 μL stop solution to terminate the reaction. Finally, the absorbance (OD) value was calculated at 650 nm using the microplate reader (Bio-Rad, USA).

### Pathological Examination

As was previously stated, all the living pigs were euthanized at 14 d.p.c., then inspected *via* macroscopical and microscopical examinations (15). We dissected lung, tonsil, and brain tissue samples and processed them with 4% formalin fixation and paraffin embedding. The samples were then prepared into 4-μm sections, followed by an H&E staining to conduct a histopathological analysis. A veterinary pathologist was invited to assess any gross pathological lesions, then histopathological examinations were conducted on selected tissue samples according to previous descriptions (18).

### Virus Shedding Detection

In line with Wang et al. (2020), viral shedding was detected. After being passaged by trypsinization, the 1 × 10^4^ Vero cells were inoculated per well in a microtiter 96-well plate (Costar, USA) in the 250 μL medium, then cultured under 5% CO_2_ and 37°C conditions until 90% confluence was achieved. When the animals were challenged, oropharynx and rectum swab samples were gathered from each pig on a daily basis to monitor virus shedding *via* TCID_50_ determination (16). After the samples were frozen and thawed three times, they were sieved through filters (0.45 μm) and diluted to 1 × 10^−1^, 1 × 10^−2^, 1 × 10^−3^, 1 × 10^−4^, and 1 × 10^−5^. The microtiter plates, which have eight wells per dilution, were seeded with diluted samples (50 μL). After a 60-min incubation at 37°C in the wells, the samples were disposed of. After they were washed three times with 0.01 M PBS (pH 7.4), the wells were filled with DMEM supplemented with 2% FBS. After a 72-h incubation, the CPE counts were microscopically inspected and recorded. The Reed-Muench method was employed to determine the TCID_50_ in each sample (16).

### Virus Load Determination in Porcine Tissues in Post-challenge Period

Autopsies were performed on the dead and surviving pigs that were terminated at 14 d.p.c. To determine virus loads, 0.1 g of brain, tonsil, and lung tissues were collected from each pig, and the viral loads in the three types of tissues were detected using the aforementioned processes (14). To briefly summarize, 0.1 g of tissue was added with DMEM (1 mL) without FBS, and the samples were frozen and thawed for three cycles and centrifugated at 5,000 × g for 10 min to homogenize. As stated in the previous section, the supernatant was indispensable when identifying the viral concentrations (TCID_50_).

### Statistical Analysis

All data are exhibited in the form of a means ± standard deviations (SD). The different tissue viral loads and viral shedding of the vaccinated-and-challenged pigs and the challenge-only pigs were investigated using a Student *t*-test and Prism software (v. 7.01; GraphPad, CA, USA); the difference was considered to be statistically significant when *p* < 0.05.

## Results

### Specific Porcine Antibody Responses in Bartha-K61 and rPRV/XJ5-gI–/gE–/TK– Vaccine Groups

**Figure 1:**
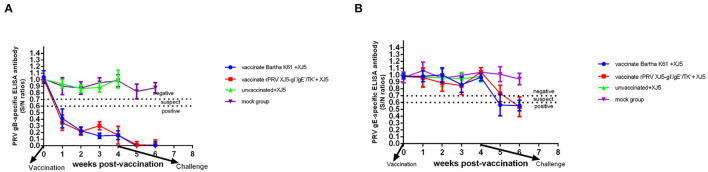
Anti-PRV sero-conversion of pigs before and after challenge; commercial ELISA kits were used to detect the anti-PRV gB antibody **(A)** and the anti-PRV gE antibody **(B)**. The dashed line indicates the cutoff value. S/N ratio = sample OD_650nm_ divided by negative control OD_650nm_; S/N < 0.6, positive; 0.6 < S/N ≤ 0.7, suspect; S/N > 0.7, negative.

In the first week post-vaccination, among the pigs vaccinated with either rPRV/XJ5-gI^−^/gE^−^/TK^−^ prototype vaccine or Bartha-K61, there was PRV gB-specific antibody that remained positive until they were challenge (Figure 1A). In contrast, there was no gE-specific antibody in the pigs from all other groups prior to being challenge, which was as expected (Figure 1B). At 0 d.p.c., the level of anti-gB antibody of the Bartha-K61-vaccinated pigs resembled that of the pigs vaccinated with rPRV/XJ5-gI^−^/gE^−^/TK^−^ (Figure 1A). Moreover, gE-specific antibodies were detected in the pigs vaccinated with either vaccine, then challenged with vPRV/ XJ-5 strain in Week 1 following the challenge (Figure 1B). All the challenge-only pigs died within 7 d.p.c.

### Observation of Clinical Symptoms After Challenge

**Figure 2:**
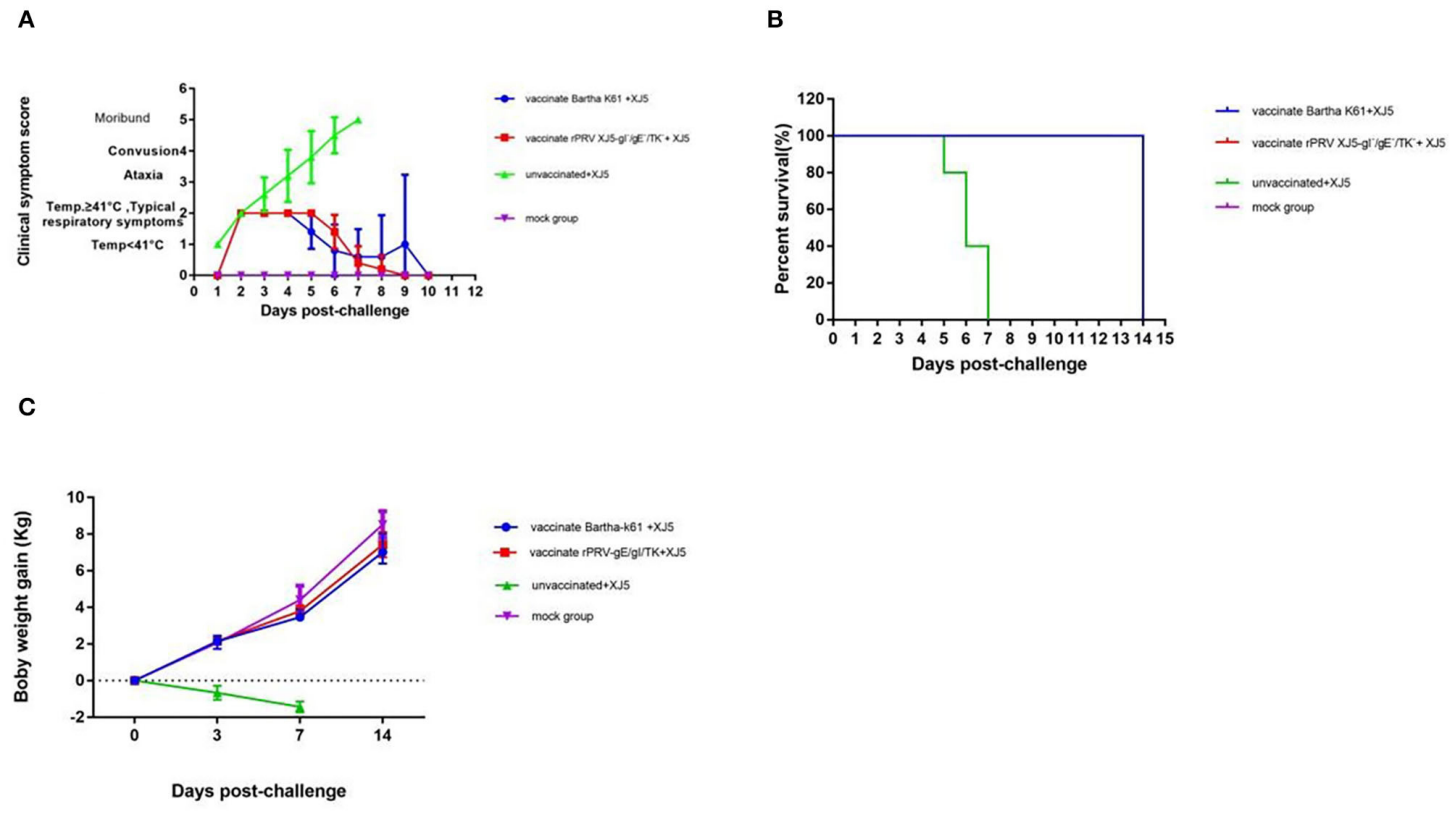
Clinical Symptoms and weight gain After Challenge. Clinical scores **(A)**, survival rate **(B)** and weight gain in different groups **(C)** (error bars represent standard errors of the mean of five replicates).

After the intranasal challenge of the PRV-free pigs, all the challenge-only pigs ran a high fever at 2 d.p.c., with body temperatures ranging from 41– 42.5°C. The documented clinical symptoms were in accordance with typical pseudorabies syndrome and included listlessness, anorexia, and high fever. Then, respiratory symptoms (i.e., coughing and sneezing) and central nervous system (CNS) symptoms emerged (Figure 2A). Notably, all pigs in the challenge-only group died within 7 d.p.c. (Figure 2B). Despite observing similar symptoms (i.e., fever and inappetence) in the Bartha-K61 group and the rPRV/XJ5-gI^−^/gE^−^/TK^−^ group, the clinical signs were more moderate, and onset occurred 1 day later than those in the challenge-only group (Figure 2A). In contrast, pigs in the mock group showed no abnormal symptoms and remained in good health (Figures 2A,B).

### Weight Gain

After the vPRV/XJ-5 challenge, the pigs in the two vaccinated groups and the mock group displayed no loss of body weight, but the challenge-only group continued losing weight until death (Figure 2C). Weight gain in the pigs vaccinated with the Bartha-K61 vaccine and pigs vaccinated with the rPRV/XJ5-gI^−^/gE^−^/TK^−^ prototype vaccine was similar to that in mock group (*p* = 1.1817 and *p* = 0.1737, respectively) at 0, 3, 7, 14 d.p.c. (Figure 2C).

### Pathological Examination

**Figure 3:**
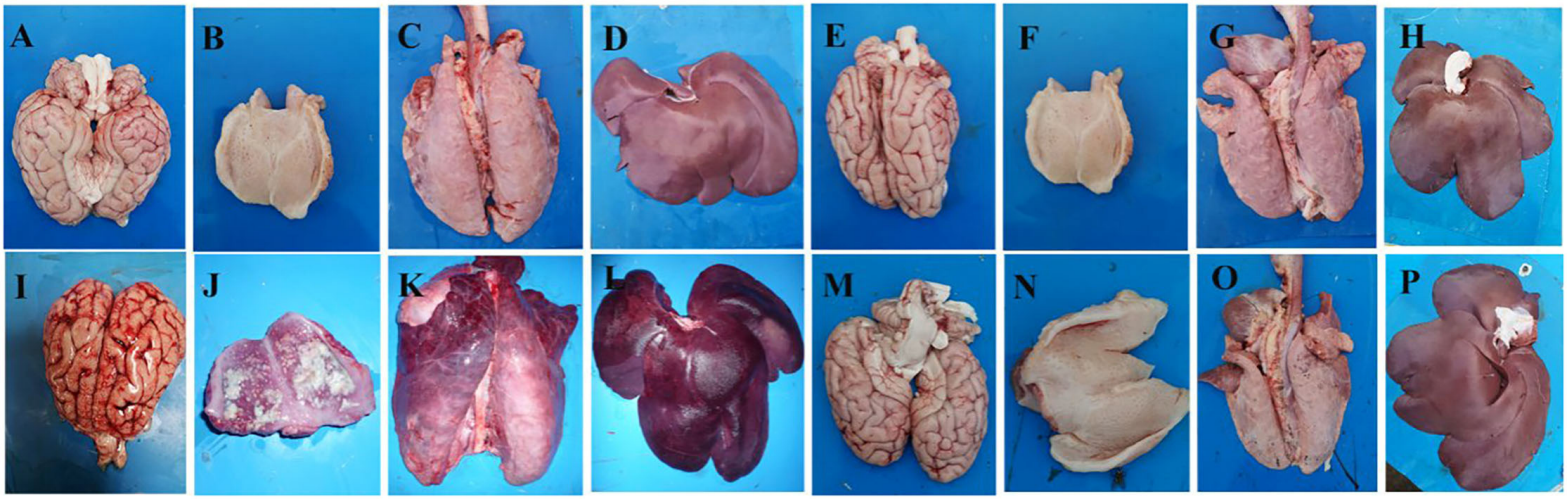
Representative gross lesions in pigs after challenge with the vPRV/XJ-5 strain. **(A–D)**: Bartha-K61 vaccinated/challenged pigs; **(E–H)**: rPRV/XJ5-gI^−^/gE^−^/TK^−^ prototype vaccine immunized/challenged pigs; **(I–L)**: Challenge-only pigs; **(M–P)**: Mock pigs; **(A,E,I,M)**: Brain; **(B,F,J,N)**: Tonsil; **(C,G,K,O)**: Lung. (**D,H,L,P**): Liver.

Autopsies were conducted on the dead pigs and the surviving pigs that were terminated at 14 d.p.c. Five of the pigs in the vPRV/XJ-5 challenged group were dead and exhibited typical lesions, cerebral edema, and congestion in the brain (Figure 3I); focal necrosis in the tonsils (Figure 3J); pulmonary congestion and edema in the lungs (Figure 3K); and focal necrosis was observed found in the liver (Figure 3L). In contrast, the tissues from animals in the mock group, the 1 × 10^6.0^ Bartha-K61-challenged group, and the rPRV/XJ5-gI^−^/gE^−^/TK^−^-challenged group did not exhibit any obvious lesions (Figure 3).

**Figure 4:**
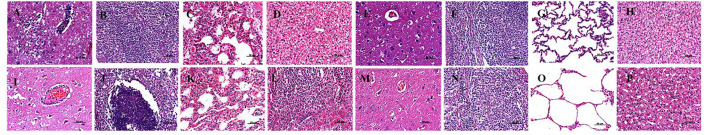
Histopathological findings in vPRV/XJ-5 strain-challenged pigs; **(A–D)**: Bartha-K61 vaccinated/challenged pigs; **(E–H)**: rPRV/XJ5-gI^−^/gE^−^/TK^−^ prototype vaccine immunized/challenged pigs; **(I–L)**: Challenge-only pigs; **(M–P)**: Mock pigs; **(A,E,I,M)**: Brain; **(B,F,J,N)**: Tonsil; **(C,G,K,O)**: Lung; **(D,H,L,P)**: Liver.

In line with these findings, a histopathological examination demonstrated that there was lymphocyte infiltration around the small vessels in the brains of the dead pigs in the vPRV/XJ-5-challenged group (Figure 4I); focal necrosis in the tonsils (Figure 4J); venous hyperemia in the lungs (Figure 4K); and focal necrosis and massive lymphocyte infiltration in the livers (Figure 4L). The Bartha-K61-vaccinated group demonstrated moderate pathological changes, and mild-to-moderate lymphocyte infiltration around the small vessels in the brain was observed (Figure 4A); lymphocyte infiltration in the alveolar, pulmonary congestion, and edema appeared (Figure 4C); and there was vacuolar degeneration in the liver (Figure 4D). In contrast, there were no pathological lesions in the tissues from the mock group and those of the pigs in the rPRV/XJ5-gI^−^/gE^−^/TK^−^-challenged group (Figure 4).

### Virus Shedding

**Figure 5:**
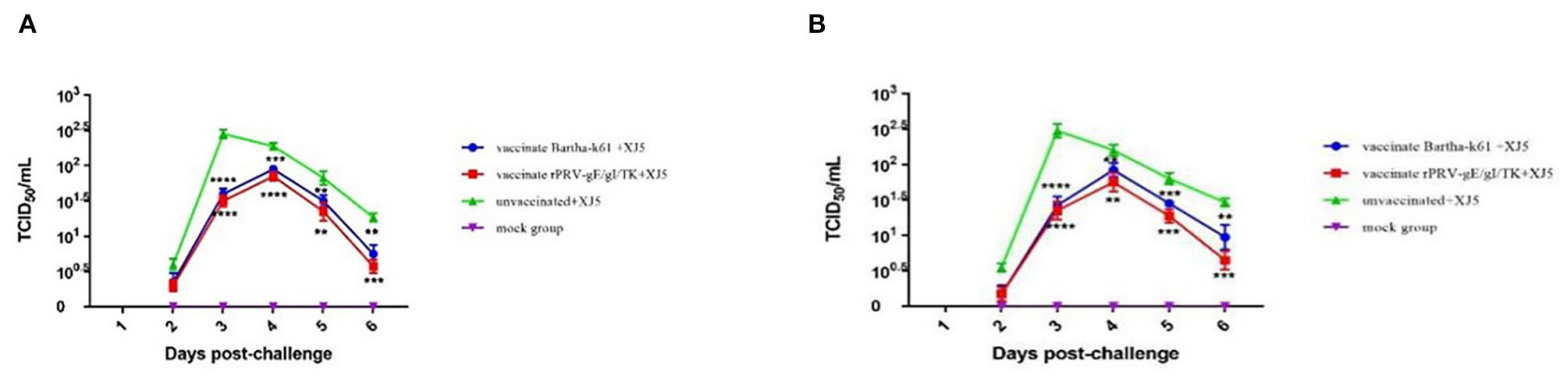
TCID_50_ of PRV in swabs from pigs in each group after challenge; **(A)** TCID_50_ of PRV in oropharyngeal swabs; **(B)** TCID_50_ of PRV in rectal swabs; (error bars represent standard errors of the mean of each group all replicates); **p < 0.01, ***p < 0.001 and ****p < 0.0001 compared with challenge-only pigs.

Virus shedding appeared in all the vPRV/XJ-5 strain-challenged pigs after 2– 6 d.p.c. (Figures 5A,B). The results indicate that the vaccination debased the amount and duration of virus shedding. The viral shedding patterns of the Bartha-K61- and rPRV/XJ5-gI^−^/gE^−^/TK^−^-immunized pigs varied from that of the challenge-only animals in the oropharyngeal swabs (Figure 5A) and rectal swabs (Figure 5B).

In the oropharyngeal swabs, the titers of the shedding virus from the pigs in the vPRV/XJ-5 challenge-only group were 1 × 10^0.60^ TCID_50_/mL at 2 d.p.c., achieved a maximum of 1 × 10^2.45^ TCID_50_/mL at 3 d.p.c., then sharp decreased to approximately 1 × 10^1.85^ TCID_50_/mL at 5 d.p.c. In contrast, the titers of the shedding virus in the vaccination groups were 1 × 10^0.30−0.35^ TCID_50_/mL at 2 d.p.c. and achieved a maximum of 10^1.85−1.95^ TCID_50_/mL at 4 d.p.c. (Figure 5A). There were noticeably fewer shedding viruses from the pigs in both vaccination groups than in the challenge-only group at 3 d.p.c. (*p* < 0.0001), and the pigs were challenged at 4 d.p.c. (*p* = 0.0001 and *p* < 0.0001, respectively). At 5 d.p.c., there were fewer shedding viruses from the pigs in both vaccination groups than there were from the challenge-only pigs (*p* = 0.0021 and *p* = 0.0010, respectively). At 6 d.p.c., the shedding viruses from the pigs in the Bartha-K61-vaccinated group were lower than those from the challenge-only group (*p* = 0.0014), and the shedding viruses from rPRVXJ5-gI^−^/gE^−^/TK^−^ prototype-inoculated pigs were significantly lower than those of the challenge-only pigs (*p* = 0.0001) (Figure 5A).

In the rectal swabs, the titers of shedding viruses from pigs in the vPRV/XJ-5 strain challenge-only group were 1 × 10^0.55^ TCID_50_/mL at 2 d.p.c., achieved a maximum 1 × 10^2.48^ TCID_50_/mL at 3 d.p.c., then rapidly declined to approximately 1 × 10^1.80^ TCID_50_/mL at 5 d.p.c. The titers of shedding viruses in the vaccination groups were 1 × 10^0.18^ TCID_50_/mL at 2 d.p.c., achieved a maximum 10^1.75−1.93^ TCID_50_/mL at 4 d.p.c., then subsequently sharply declined to approximately 1 × 10^1.28−1.45^ TCID_50_/mL at 5 d.p.c. (Figure 5B). At 3 d.p.c. (*p* < 0.0001) and 4 d.p.c. (*p* = 0.0047 and *p* = 0.0011, respectively), the shedding viruses from the pigs in the challenge group significantly exceeded those from both vaccination groups. At 5 d.p.c., the shedding viruses from pigs in the challenge-only group were noticeably higher than those from the pigs in both vaccination groups (*p* = 0.0004 and *p* = 0.0002, respectively). Notably, at 6 d.p.c., the shedding viruses from the pigs in the Bartha-K61 vaccine group did not exceed those of the challenge group (*p* = 0.0054), and there were fewer shedding viruses from the pigs in the rPRV/XJ5-gI^−^/gE^−^/TK^−^ group than was observed from the challenge-only pigs (*p* = 0.0002) (Figure 5B).

### PRV Viral Load in Pig Tissues Post-challenge

**Figure 6:**
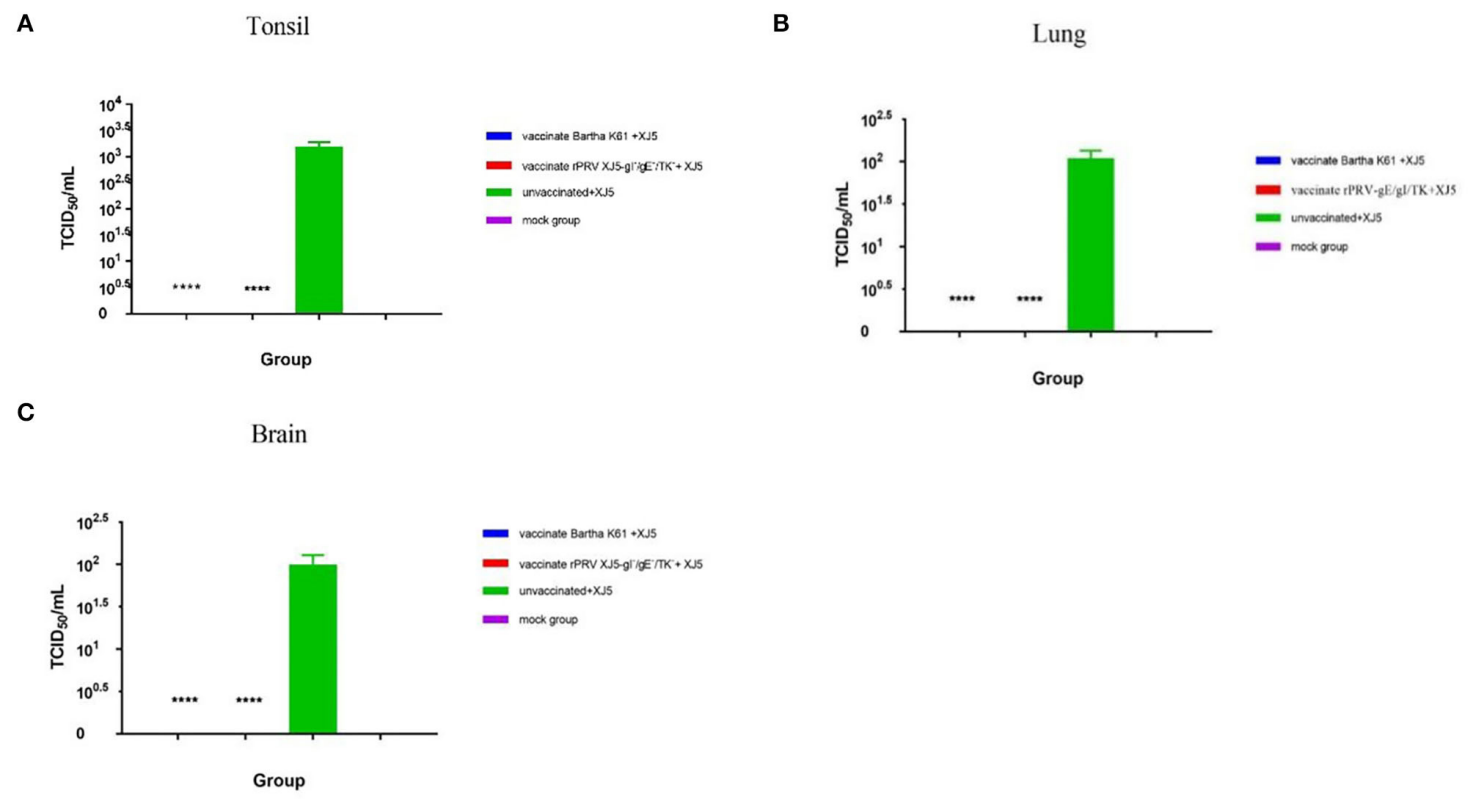
PRV viral loads in tonsil **(A)**, lung **(B)**, and brain **(C)** tissues of pigs upon necropsy at 14 d.p.c. (error bars represent standard errors of the mean of each group all replicates); ****p < 0.0001 compared with challenge-only pigs.

When the viral load of the XJ-5 strain of PRV-1 was measured in various tissues after the challenge, a viral load was not detected in any tissues from the Bartha-K61- and rPRV/XJ5-gI^−^/gE^−^/TK^−^-immunized pigs; and a high viral load was detected in the challenge-only pigs (Figures 6A–C). In the challenge-only group, a viral load of approximately 1 × 10^3.1^ TCID_50_/mL was found in the tonsils (Figure 6A); a viral load of approximately 1 × 10^2.0^ TCID_50_/mL was observed in the lungs (Figure 6B); and a viral load of approximately 1 × 10^2.4^ TCID_50_/mL was recorded in the brain (Figure 6C).

## Discussion

As a live vaccine that has been modified, the PRV Bartha-K61 strain with deleted gE and partial gI genes has been extensively used; remarkably, eliminating the gE gene attenuated the virus in nearly every species of animals that were vulnerable to PRV (19). The pigs had been previously vaccinated with the Bartha-K61 vaccine at a dose of 1 × 10^6.3^ TCID_50_ per animal and challenged with the vPRV/XJ-5 strain, when all the vaccinated piglets were protected from the lethal challenge (15). It was recently discovered that the Bartha-K61 vaccine at a dose of 1 × 10^5.0^ TCID_50_/animal protected pigs against the vPRV XJ-5 strain-induced sublethal challenge and exhibited an equivalent performance to an identical dose of the rPRV/XJ5-gI^−^/gE^−^/TK^−^ vaccine, even though it should be noted that the Bartha-K61 vaccine at lower doses alone did not provide protection against such a challenge (14).

In this study, all pigs vaccinated with either the Bartha-K61 vaccine or the rPRV/XJ5-gI^−^/gE^−^/TK^−^ prototype vaccine survived after being challenged with the XJ-5 strain of vPRV. And the clinic scores, virus shedding, weight gain, and viral loads in different tissues of pigs in these two groups were extremely similar, which suggested that the same dose of Bartha-K61 and rPRV/XJ5-gI^−^/gE^−^/TK^−^ vaccines had the same level of efficacy against a lethal challenge of the XJ-5 strain of vPRV. It was confirmed that the Bartha-K61 and rPRV/XJ5-gI^−^/gE^−^/TK^−^ prototype vaccines presented an equal level of protection in growing pigs after a lethal vPRV challenge. However, there was increasing evidence that the Bartha-K61 vaccine could not provide complete protection for piglets against these new variants (12, 20, 21)which mainly focused on the 3– 4-week-old piglets comparing the vaccine efficacy in some research (13). And growing pigs older than 12 weeks were rarely used for this purpose. Compared with previous studies, other factors such as vaccination doses and challenge doses were also significantly different (22).

In the present study, 12-week-old PRV/gB and PRV/gE antibody-free growing pigs were immunized with either the Bartha-K61 vaccine or the rPRV/XJ5-gI^−^/ gE^−^/ TK^−^ prototype vaccine at a dose of 1 × 10^6.0^ TCID_50_ per animal, after which a lethal challenge of the XJ-5 strain of vPRV was implemented. The high mortality rate of the 16-week-old challenge-only pigs in this study coincided with the clinical observations of a high fatality rate caused by vPRV among newborn, growing, and finishing pigs (i.e., 50, 10, and 30%, respectively) (12). Similar experimental evidence for a growing pig challenge model based on vPRV is scarcely available in previous studies.

For growing pigs, weight gain is a critical parameter to assess the PRV vaccine efficacy and that is associated with economic benefit (23, 24). In the present study, the challenge-only pigs experienced obvious weight loss prior to death, but weight gain in the pigs vaccinated with the Bartha-K61 vaccine and the rPRV/XJ5-gI^−^/gE^−^/TK^−^ prototype vaccine after the challenge were similar to that in the mock group. While the Bartha-K61 vaccinated group exhibited moderate pathological changes in the brain with mild-to-moderate lymphocyte infiltration around the small vessels and lungs with lymphocyte infiltration in the alveolar, pulmonary congestion, and edema appeared at 14 d.p.c., no pathological lesions were observed in the rPRV/XJ5-gI–/gE–/TK– prototype vaccine group at 14 d.p.c. For highly virulent vPRV strains, vPRV with double gE-and-gI gene deletions resulted in pathological lesions in the brain and lung tissues of both the mice and the piglets (25). It was also found that the double gene-deletion-based rPRV/XJ5-gI–/gE– strain and the triple gene-deletion-based rPRV/XJ5-gI–/gE–/TK– strain stayed virulent in mice. Based on these findings, triple TK/gE/gI-gene-deleted recombinant vPRV demonstrated a higher level of safety in the candidate swine gene-deletion pseudorabies virus variant vaccines than the double gE/gI-gene-deleted vPRV (26).

The DNA vaccination against PRV was shown to be deficient to a certain extent, because virus excretion must be effectively reduced during the first week after the pigs are infected (27). Based on the current study, regardless of whether they were vaccinated, virus shedding was observed in all challenged pigs; this suggests that the Bartha-K61 vaccine is unable to protect challenged pigs against viral excretion, which confirms when pigs are challenged with PRV/TJ, the Bartha-K61 vaccine cannot prevent viral shedding as was concluded in a previous study (28). Furthermore, the same situation existed in rPRV/XJ5-gI^−^/gE^−^/TK^−^-vaccinated pigs during the first week after the challenge, which implied that neither the Bartha-K61 vaccine nor the rPRV-gI^−^/gE^−^/TK^−^ vaccine were able to guard against viral shedding when challenged with PRV. Moreover, it should also be noted that during the post-challenge period, the shedding viruses in the oropharyngeal swabs of the Bartha-K61 and rPRV/XJ5-gI^−^/gE^−^/TK^−^ prototype-vaccinated pigs were the same as to those in the rectal swabs when the pigs were similarly vaccinated. Based on the necropsy on the 14 d.p.c., the highest viral load was found in the tonsils, followed by brains and lungs, which coincided with the development of CNS and respiratory symptoms in the challenged pigs.

In conclusion, the findings of this study suggest that a 1 × 10^6.0^ TCID_50_ dose of the Bartha-K61 vaccine can efficiently protect growing pigs from a lethal challenge of the vPRV XJ-5 strain, although this efficacy was little lower than that of the same dosage of the rPRV/XJ5-gI^−^/gE^−^/TK^−^ prototype vaccine. The Bartha-K61 vaccine can therefore be regarded as an efficient tool to fight the porcine pseudorabies caused by vPRV in China. The efficacy of prototype rPRV vaccines (e.g., rPRV/XJ5-gI^−^/gE^−^/TK^−^) against different kinds of vPRV strains challenge, especially compared to an equal dosage of the Bartha-K61 vaccine, requires further study.

## Data Availability Statement

The original contributions presented in the study are included in the article/supplementary material, further inquiries can be directed to the corresponding authors.

## Ethics Statement

The animal study was reviewed and approved by Yangzhou University [approval ID: SYXK (Su) 2007–0005].

## Author Contributions

QR and LL: conceptualization and writing—original draft preparation. HP: methodology and data validation and formal analysis. CH and YK: investigation. SG: resources and funding acquisition. CC: writing—review and editing. All authors have read and agreed to the published version of the manuscript.

## Funding

This study was funded by grants from the Key R&D Program of Jiangsu Province (BE2020320), the National Key R&D Program (2016YFD0500704-2), the Novel Agricultural Research Program of Jiangsu Province [SXGC(2017)231], the funding from the Priority Academic Program Development of Jiangsu Higher Education Institutions (PAPD), and Science and Technology Support Program of Jiangsu Province (BE2014355) as well as the earmarked fund for Jiangsu Agricultural Industry Technology System [JATS(2018)221].

## Conflict of Interest

The authors declare that the research was conducted in the absence of any commercial or financial relationships that could be construed as a potential conflict of interest.

## Publisher's Note

All claims expressed in this article are solely those of the authors and do not necessarily represent those of their affiliated organizations, or those of the publisher, the editors and the reviewers. Any product that may be evaluated in this article, or claim that may be made by its manufacturer, is not guaranteed or endorsed by the publisher.
